# An Electrochemical Sensor Based on Electropolymerization of β-Cyclodextrin on Glassy Carbon Electrode for the Determination of Fenitrothion

**DOI:** 10.3390/s23010435

**Published:** 2022-12-30

**Authors:** Rong Wang, Shulong Wang, Caihong Qin, Qiyang Nie, Yougang Luo, Qi-Pin Qin, Ruijuan Wang, Baiquan Liu, Dongxiang Luo

**Affiliations:** 1 Guangxi Key Lab of Agricultural Resources Chemistry and Biotechnology, College of Chemistry and Food Science, Yulin Normal University, 1303 Jiaoyudong Road, Yulin 537000, China; 2 State Key Laboratory of Luminescent Materials and Devices, South China University of Technology, 381 Wushan Road, Guangzhou 510640, China; 3 School of Electronics and Information Technology, Sun Yat-sen University, Guangzhou 510275, China; 4 Huangpu Hydrogen Innovation Center, School of Chemistry and Chemical Engineering, Guangzhou University, Guangzhou 510006, China

**Keywords:** electrochemical polymerization, β-cyclodextrin, pesticide determination, fenitrothion

## Abstract

An electrochemical sensor enabled by electropolymerization (EP) of β-cyclodextrin on glassy carbon electrode (β-CDP/GCE) is built for the determination of fenitrothion (FNT). The effects of the EP cycles, pH value, and enrichment time on the electrochemical response of FNT were studied. With the optimum conditions, good linear relationships between the current of the reduction peak of the nitroso derivative of FNT and the concentration are obtained in the range of 10–150 and 150–4000 ng/mL, with a detection limit of 6 ng/mL (S/N = 3). β-CDP/GCE also exhibits a satisfactory applicability in cabbage and tap water, with recovery values between 98.43% and 112%. These outstanding results suggest that β-CDP/GCE could be a new effective alternative for the determination of FNT in real samples.

## 1. Introduction

Nowadays, pesticides have been widely used to increase the yield of crops, which has made pesticide pollution a major issue of global concern [1,2]. Fenitrothion (FNT) is one of the organophosphorus insecticides, and is widely used in crops such as grain, cotton, and fruit. However, FNT is acutely toxic and can cause severe effects on the central nervous system; thus, its pollutants are harmful to animals, the environment, and human beings [3,4,5]. Therefore, it is very important to detect residues of FNT in food samples.

Traditional pesticide-detection methods include chromatography and spectroscopic techniques such as gas chromatography, liquid chromatography, gas chromatography–mass spectrometry, fluorescence spectra, etc. [6,7,8,9,10]. These methods have high sensitivity, and are very suitable for laboratory detection. Bioassays such as immunoassays and enzyme inhibition are widely accepted as rapid detection methods [11,12,13,14]. Electrochemical detection technology has the advantages of high sensitivity, accuracy, portability of equipment, simple operation, fast analysis speed, and low cost, and thus it has gradually become a research hotspot in the field of pesticide detection [15,16,17,18].

Due to the development of materials science, a number of materials have been used as electrode modifiers to improve the performance of pesticide detection, such as graphene [19], multi-carbon nanotubes [20], metal nanoparticles [21], conductive polymers [22], β-cyclodextrin (β-CD) [23], etc. β-CD is a naturally occurring cyclic oligosaccharide consisting of seven glucose units, which presents a toroidal shape with an inner hydrophobic cavity and an outer hydrophilic shell. This special structure enables it to incorporate molecules with suitable size to form stable inclusion complexes [24,25]. A β-CD modifier could improve the reversibility of the electrode reaction, facilitate the electron transfer, and increase the selectivity. Therefore, β-CD has been widely studied as an electrode modifier for the selective determination of various electroactive molecules, such as neonicotinoids [26], gatifloxacin [27], nitroaromatic isomers [28], dopamine, uric acid [29], etc. However, to the best of our knowledge, there are few studies using β-CD as a modifier for FNT determination.

In this work, aiming to explore the unrevealed sensor potential, an electrochemical sensor for FNT determination is prepared via electropolymerization (EP) of β-CD onto GCE (β-CDP/GCE). The inner cavities of β-CDP could increase the surface of the electrode and accumulate the analytes at the electrode. The response current of FNT is greatly enhanced on β-CDP/GCE. With optimized experimental conditions, the developed sensor exhibits good performance and is successfully applied for quantitative determination of FNT in cabbage and tap water samples with satisfactory results.

## 2. Materials and Methods

### 2.1. Reagents and Apparatus

FNT (100.000%) was obtained from Accustandard (America). β-CD (98%) was purchased from Macklin (Shanghai, China). Na_2_HPO_4_·12H_2_O, NaH_2_PO_4_·2H_2_O, CH_3_COOH, CH_3_COONa·3H_2_O, KCl, NaCl, and ZnCl_2_ were of analytical grade and purchased from Xilong Scientific Co., Ltd. (Shantou, China). K_3_[Fe(CN)_6_] and K_4_[Fe(CN)_6_] were purchased from Tianjin Guangfu Technology Development Co., Ltd., (Tianjin, China). All reagents were used without further purification. Phosphate buffer solution was prepared with 0.1 M Na_2_HPO_4_·12H_2_O and NaH_2_PO_4_·2H_2_O. Acetate buffer solution was prepared with 0.1 M CH_3_COOH and CH_3_COONa·3H_2_O. Purified water (Wahaha Group Co., Ltd., Hangzhou, China) was used throughout.

All electrochemical experiments were finished with a CHI660E electrochemical workstation (Shanghai Chenhua Instrument Co., Ltd., Shanghai, China) with a three-electrode system. GCE (diameter = 3 mm) or β-CDP/GCE was used as the working electrode, a platinum wire as the auxiliary electrode, and a saturated calomel electrode (SCE) as the reference electrode. The pH of the buffer solution was monitored with a pH meter (Leici PHSJ-3F, Shanghai Yishan Scientific Instrument Co., Ltd., Shanghai, China).

### 2.2. Preparation of β-CDP/GCE

The GCE was first polished with 0.05 micron alumina and washed with distilled water. Then the polished GCE was immersed in a mixed solution of 1 M KCl containing 5 mM K_3_[Fe(CN)_6_], and an electrochemical test was performed with cyclic voltammetry (CV) with a potential from 0.5 to −0.1 V, scan rate of 50 mV/s, and 1 cycle. If the potential difference between the reduction and the oxidation peak of [Fe(CN)_6_]^3−^/[Fe(CN)_6_]^4−^ was less than 100 mV, it indicated that the GCE was clean. Otherwise, it was necessary to repeat the above operation until it was less than 100 mV. Finally, the GCE was ultrasonicated in ethanol for 1 min and dried in air.

β-CD solution was prepared by mixing 6 mM β-CD in 0.1 M phosphate buffer solution (pH = 6.80). GCE was immersed in the β-CD solution and the EP process was performed using the CV method with the potential from −2.0 to 2.0 V, scan rate of 100 mV/s, and 10 consecutive cycles. Then the formed β-CDP/GCE was treated in 0.1 M phosphate buffer solution (pH = 6.80) using the CV method with a potential from −2 to 2.0 V, scan rate of 100 mV/s, and 3 cycles. Finally, the β-CDP/GCE was rinsed with purified water, dried in air, and stored at room temperature.

### 2.3. Electrochemical Impedance Spectroscopy Measurements

Electrochemical impedance spectroscopy (EIS) was performed in 5.0 mM K_3_[Fe(CN)_6_]/K_4_[Fe(CN)_6_] (1:1) containing 0.1 M KCl. The initial potential was set as the open circuit potential and the amplitude was 5 mV. The frequency range was 10 to 10^3^ Hz for bare GCE and 10^−1^ to 10^3^ Hz for β-CDP/GCE.

### 2.4. Preparation and Determination of Cabbage and Tap Water Samples

The cabbage was purchased from a local vegetable market, ground with a mortar, and filtered to obtain the cabbage juice. Tap water was taken directly from the laboratory. FNT was added to 1 mL of the above cabbage juice or tap water, then the mixture was diluted 10-fold with acetate buffer (pH = 5.00), making the added FNT concentrations 50 ng/mL and 2000 ng/mL, respectively. β-CDP/GCE was used as the working electrode, and the differential pulse voltammetry (DPV) method with a three-step scan was performed. According to the peak current of the third-step DPV curve and calibration curve, the theoretical concentration of FNT was calculated. The recovery rate was the ratio between the theoretical concentration and the actual added concentration.

## 3. Results and Discussion

### 3.1. Electrochemical Behavior of Electropolymerized β-CD on GCE

**Figure 1:**
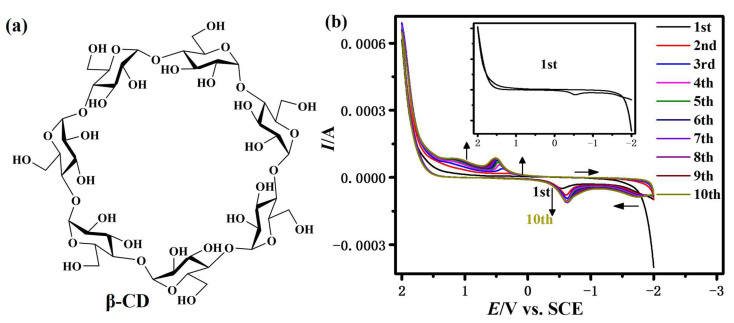
(**a**) The chemical structure of β-CD; (**b**) multicycle CV curves of β-CD (6 mM in phosphate buffer solution, pH = 6.80) on GCE with potential from −2.0 to 2.0 V, scan rate of 100 mV/s and 10 cycles. Inset: the first CV curve of β-CD on GCE.

The chemical structure of β-CD is shown in Figure 1a, which is a cyclic oligosaccharide consisting of seven glucose units. CV was chosen as the EP method, which could well control the deposition rate through adjusting the EP parameters [30]. Figure 1b presents the first ten cycles’ CV curves of β-CD on GCE. During the first cycle, the current gradually increases at about 1.5 V, which is attributed to the oxidation of β-CD generating free radical cations. The free radical cations couple with each other to form polymers and the polymers are deposited on the electrode surface. A reduction peak occurs at about −0.55 V, which may be caused by the reduction of β-CD polymers (β-CDP) deposited on the electrode. From the second cycle, two new oxidation peaks are observed at about 0.45 and 1.1 V; both could be attributed to the oxidation of β-CDP deposited on the electrode. In the subsequent scan, the peak current (−0.55, 0.45, and 1.1 V) gradually increases with the increase in cycle number, suggesting the growth of a β-CDP film on the surface of GCE to form the β-CDP/GCE-modified electrode. According to Faraday’s law, the quantity of the deposited β-cyclodextrin was calculated as about 0.1 μg/mm^2^ after ten cycles. This electrochemical behavior of β-CD is similar to that observed in other reports [29]. Figure S1 illustrates the surface morphology of bare GCE and β-CDP/GCE. Compared with bare GCE, β-CDP completely covers the surface of electrode with a structure of spherical particles, which confirms that β-CDP was successfully deposited on GCE. The EIS reveals the impedance change of the electrode and provides detailed information on the surface properties of the electrode [27]. The EIS measurements of bare GCE and β-CDP/GCE are shown in Figure S2. The electron transfer resistance increases after modification with β-CDP, which would make charge transfer difficult. This is mainly caused by the nonconducting β-CDP films deposited on GCE [23,27].

### 3.2. Electrochemical Response of FNT

**Figure 2:**
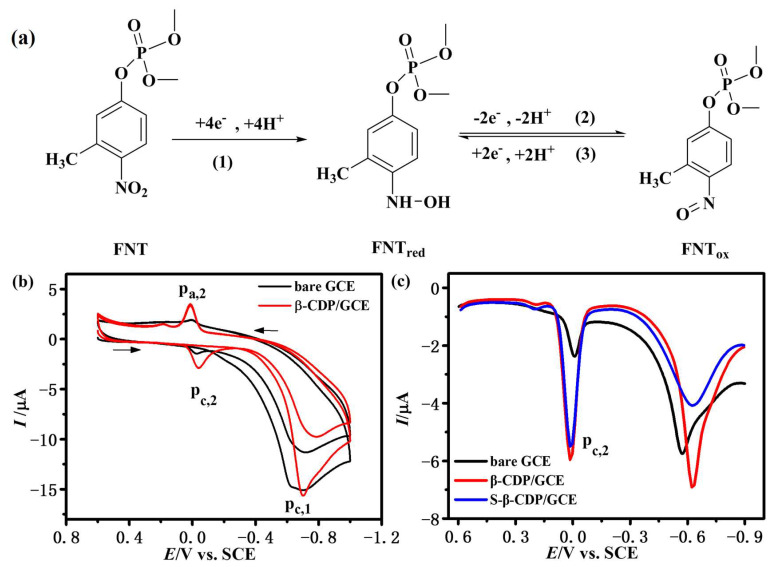
(**a**) The chemical structure of FNT and its electrochemical behavior; (**b**) CV curves of FNT (2 μg/mL in acetate buffer solution, pH = 5.00) on GCE and β-CDP/GCE with potential from 0.6 to −1.0 V, scan rate of 50 mV/s, and 2 cycles; (**c**) the third-step DPV curves of FNT (2 μg/mL in acetate buffer solution, pH = 5.00) on GCE (black line) and β-CDP/GCE (red line); (blue line) the third-step DPV curve of S-β-CDP/GCE (soaked in acetate buffer solution with 2 μg/mL FNT for 90 s, taken out, and rinsed with acetate buffer solution) in blank (without FNT) acetate buffer solution (pH = 5.00). DPV parameters: potential increment of 13 mV, amplitude of 50 mV, pulse width of 60 ms, sampling width of 20 ms, pulse period of 500 ms, and potential from 0.6 to −0.9 V.

The chemical structure of FNT is shown in Figure 2a; it contains an electro-active nitro (–NO_2_) group. Its electrochemical behavior and the first two CV curves are shown in Figure 2a,b. During the first cycle, a reduction peak appears at about −0.6 V (p_c,1_), which is attributed to the reduction of –NO_2_ into hydroxylamine (–NHOH), resulting in the formation of FNT_red_, as shown in reaction (1). Then an oxidation peak at about 0.01 V (p_a,2_) occurs, which belongs to the oxidation of –NHOH of FNT_red_ into nitroso (–NO), forming FNT_ox_, as presented in reaction (2). In the second cycle, a new reduction peak at about −0.03 V (p_c,2_) is observed, which is caused by the reduction of –NO of FNT_red_ into –NHOH, as shown in reaction (3). Reaction (2) and (3) are reversible. These results are consistent with the literature [30]. The peak currents of p_c,1_, p_a,2_, and p_c,2_ (*I*_pc,1_, *I*_pa,2_, and *I*_pc,2_, respectively) are proportional to the concentration of FNT, which could be used for the quantitative analysis of FNT. The electrochemical response of FNT on β-CDP/GCE is similar to GCE, but the current has a great enhancement. However, the electrochemical behavior of β-CDP itself interferes with the *I*_pc,1_ and *I*_pa,2_ of FNT; thus we choose *I*_pc,2_ for the quantitative analysis. The effect of scan rate on the *I*_pc,2_ at β-CDP/GCE is shown in Figure S3. The *I*_pc,2_ varies linearly with the scan rates from 10 to 300 mV/s, which indicates a typical adsorption-controlled process [27].

Usually, pulse voltammetry can effectively avoid the influence of charging current, and thus give a higher sensitivity. However, *I*_pc,2_ is generated in reaction (3), hence we need a three-step scan when using the DPV method. In the first step (from 0.6 to −0.9 V), FNT is reduced to FNT_red_, then FNT_red_ is oxidized to FNT_ox_ in the second step (from −0.9 to 0.6 V), and FNT_ox_ is reduced to FNT_red_ again in the third step (from 0.6 to −0.9 V). Finally, *I*_pc,2_ in the third step is adopted for the quantitative analysis of FNT. Figure S4 shows the electrochemical response of FNT with theCV and DPV methods. The *I*_pc,2_ with the DPV method is higher than the CV method, and gives a smoother baseline and a more positive potential. Therefore, the DPV method was chosen for the subsequent experiments. However, the third-step DPV curve of β-CDP/GCE in blank (without FNT) acetate buffer shown in Figure S5 (black line) reveals that there is strong current around 0 V, which would seriously interfere with the quantitative analysis of FNT. This may be caused by the reduction reaction of β-CD monomer and β-CDP cation doped in β-CDP film. This interference can be effectively eliminated (as shown in Figure S5 (red line)) after immersing β-CDP/GCE in blank (without β-CD) phosphate buffer solution and treating with the CV method with a potential from −2 to 2.0 V, scan rate of 100 mV/s, and three cycles. Figure 2c also compares the electrochemical response of FNT on bare GCE and β-CDP/GCE. The faradaic current on β-CDP/GCE is significantly larger than bare GCE, which is mainly attributed to the good recognition and enrichment ability of β-CDP. A larger current would lead to higher sensitivity and improve the determination performance. To investigate the interaction of FNT with β-CDP, β-CDP/GCE is soaked in acetate buffer solution (pH = 5.00) with 2 μg/mL FNT for 90 s, taken out and rinsed with acetate buffer solution, and marked as S-β-CDP/GCE. As shown in Figure 2c, the *I*_pc,2_ of S-β-CDP/GCE in blank (without FT) acetate buffer solution (pH = 5.00) is close to the β-CDP/GCE in acetate buffer solution (pH = 5.00) with 2 μg/mL FNT, which indicates that FNT is included in β-CDP cavities.

### 3.3. Optimization of Experimental Conditions

#### 3.3.1. Effect of EP Cycles

**Figure 3:**
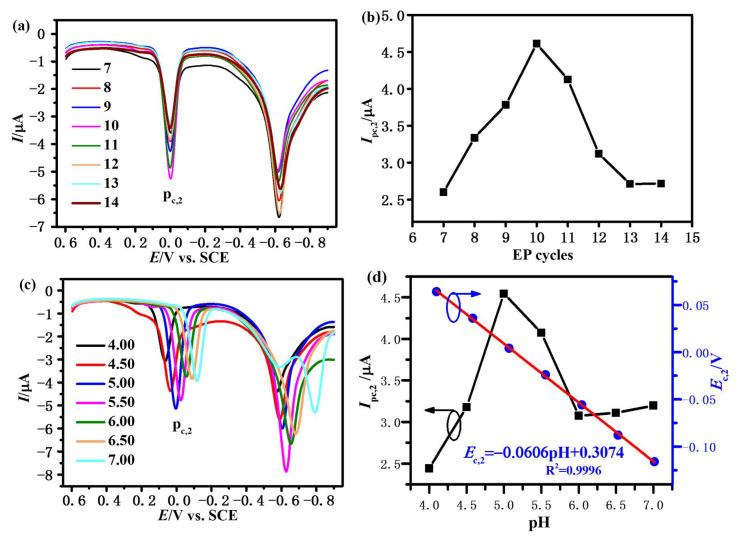
(**a**) The third-step DPV curves of FNT (2 μg/mL in acetate buffer solution, pH = 5.00) on β-CDP/GCE prepared with different EP cycles; (**b**) the variation in *I*_pc,2_ with EP cycles; (**c**) the third-step DPV curves of FNT (2 μg/mL in acetate buffer solution with different pH) on β-CDP/GCE; (**d**) the variation in *I*_pc,2_ and *E*_c,2_ with pH. DPV parameters: potential increment of 13 mV, amplitude of 50 mV, pulse width of 60 ms, sampling width of 20 ms, pulse period of 500 ms, and potential from 0.6 to −0.9 V.

The thickness of β-CDP film is determined by the EP cycles, which would affect its electrochemical activity. The third-step DPV curves of FNT on β-CDP/GCE prepared with different EP cycles and the variation of *I*_pc,2_ with EP cycles are shown in Figure 3a,b. The *I*_pc,2_ increases with EP cycles from seven to ten, which demonstrates that the recognition and enrichment ability of β-CDP to FNT is increased with EP cycles. However, *I*_pc,2_ decreases when there are more than ten cycles, indicates that redundant scans would lead to a decrement in conductivity and hinder the electron transfer. As a consequence, the EP cycle of ten provides the best result.

#### 3.3.2. Effect of pH

The influence of the pH value of the buffer solution was also investigated, as illustrated in Figure 3c,d. The *I*_pc,2_ rises as the pH value increases, then reaches a highest value at pH = 5.00, but reduces after 5.00. Consequently, pH = 5.00 was chosen for electrochemical measurements, which is well consistent with other reports in the literature [31].

Moreover, Figure 3d also demonstrates that the potential of p_c,2_ ($E_{c,2}$) also depends on the pH and shifts to negative values with pH increases. $E_{c,2}$ is linearly related to pH, which is due to the participation of protons in reaction (3).

$$FNT_{ox}+2e^{-}+mH^{+}⇌FNT_{red}$$

where **m** is the number of protons. According to Nernst’s equation, $E_{c,2}$ can be given as the below equation:

$$E_{c,2}=E_{c,2}^{⊖}-\left(\frac{2.303mRT}{2F}\right)\mathrm{pH}$$

where $E_{c,2}^{⊖}$ is the standard electrode potential of reaction (3), *R* is the molar gas constant (8.314 J·mol^−1^·K^−1^), *F* is the Faraday constant (96,500 C·mol^−1^), and *T* is the temperature. As shown in Figure 3d, the slope of the potential–pH diagram is 0.0606 V/pH, which is in agreement with the theoretical slope (2.303 m*RT*/2*F*) of 0.059 V/pH with T = 298 K and m about 2. These results indicate that reaction (3) is a two-electron and two-proton process, as described in Figure 2a. We also measured the pH of the solution before and after the reaction, and found that there is no change in the pH, which demonstrates that the pKa of FNT_red_ would not affect the result. This is mainly because the amount of FNT_red_ reacted on the electrode surface is very small (according to Faraday’s law, only about 0.205 ng/mm^2^ when the FNT concentration is 2 μg/mL).

#### 3.3.3. Accumulation Time

The relationship between the *I*_pc,2_ and the accumulation time of every step in the DPV method was studied. As shown in Figure S6, the *I*_pc,2_ reached its maximum values with accumulation at open circuit of 90 s for the first and second step and 60 s for the third step. These results indicate that more recognition and enrichment between β-CDP and FNT occur with the increase in accumulation time. However, the combination would reach saturation with enough time.

#### 3.3.4. Parameters of DPV Method

Figure S7 illustrates the impact of the parameters (potential increment and amplitude) of the DPV method on the electrochemical response of FNT. The *I*_pc,2_ increases first and then decreases with the increase in potential increment, and reaches its maximum at 4 mV. The case of amplitude is also similar to that of potential increment, and is best at 50 mV. Therefore, the parameters of the DPV method are optimized as follows: potential increment of 13 mV, amplitude of 50 mV, pulse width of 60 ms, sampling width of 20 ms, pulse period of 500 ms.

### 3.4. Calibration Curve

**Figure 4:**
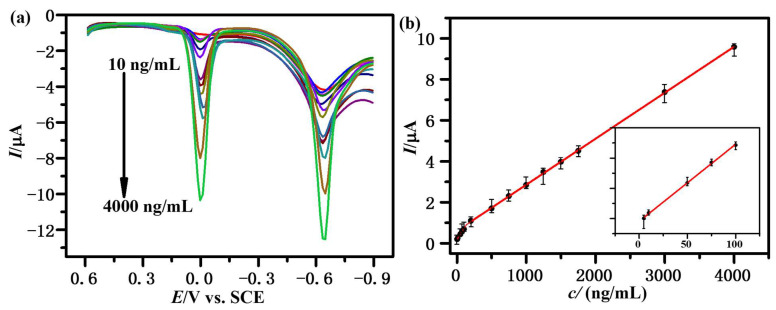
(**a**) The third-step DPV curves of FNT with different concentrations (10, 50, 75, 100, 200, 500, 750, 1000, 1250, 1500, 1750, 3000, 4000 ng/mL in acetate buffer solution, pH = 5.00) on β-CDP/GCE; (**b**) the calibration curve for *I*_pc,2_ versus concentration of FNT.

Under the above optimum conditions, we obtained the third-step DPV curves of FNT with different concentrations and the corresponding calibration curve. As shown in Figure 4, the *I*_pc,2_ are proportional to the concentration of FNT in the range 10–150 ng/mL and 150–4000 ng/mL with a limit of detection of 6 ng/mL (S/N = 3) and limit of quantification of 10 ng/mL. The equations of the calibration curves are I_1_(μA) = 0.00501*c* (ng/mL) + 0.1881 (R^2^ = 0.9991) and I_2_(μA) = 0.00225*c* (ng/mL) + 0.6107 (R^2^ = 0.99985), respectively. This excellent result is related to the modifier of β-CDP, which may recognize and enrich FNT molecules on the electrode. The comparisons of the proposed β-CDP/GCE with other electrochemical sensors reported previously are displayed in Table 1, in which the proposed β-CDP/GCE shows a satisfying result. These demonstrate that β-CDP/GCE would be a promising electrochemical sensor for FNT determination.

### 3.5. Repeatability and Interference

Repeatability and interference are important parameters to evaluate the reliability of a method. In order to investigate the repeatability of the proposed sensor, seven sensors were fabricated in the same manner. The results shown in Figure S8 give a relative standard deviation of 4.09%, which indicates that the proposed sensor has a good repeatability. The interference was studied by evaluating the effect of various possible interfering compounds such as K^+^, Na^+^, Zn^2+^, imidacloprid (IDP), etc. As shown in Figure S8, no interference was found with 2000-fold of KCl, NaCl, and ZnCl_2_, which suggests that the β-CDP/GCE has excellent anti-interference ability for inorganic salts. However, there is some interference with IDP, mainly because it has the same active group, a nitro group.

### 3.6. Real Sample Analysis

In order to evaluate the practical application of the proposed electrode, cabbage and tap water samples were tested, and no FNT was found in the samples. Furthermore, a standard-addition method was used for FNT analysis of cabbage and tap water. As presented in Table 2, the recovery values are 99.4% and 107.25% in cabbage samples, and 98.34% and 112% in tap water. These results indicate a promising applicability of β-CDP/GCE in real samples [36,37,38].

## 4. Conclusions

An electrochemical sensor based on electropolymerized β-CD was built for FNT determination. Under the optimum conditions, in the ranges of 10–150 and 150–4000 ng/mL, good linear relationships between the current of the reduction peak of the nitroso derivative of FNT and the concentration are obtained, with a detection limit of 6 ng/mL (S/N = 3). β-CDP/GCE also exhibits a satisfactory applicability in cabbage and tap water, with recovery values between 98.43% and 112%. These excellent results are mainly attributable to the recognition and enrichment of β-CDP, and suggest that β-CDP/GCE provides a novel, simple, yet rapid approach for the determination of FNT.

## Figures and Tables

**Table 1:** Comparison of different electrochemical sensors for the determination of FNT.

Sensors	Linear Ranges (μM)	Detection Limit (μM)	Ref.
MWCNT/GCE	0.2–60	0.08	[31]
CoPc(ma)/GCE	1.2–42.0	0.46	[32]
GO/GCE	0.0036–1.44	0.1	[33]
CeO_2_@RGO/GCE	0.025–2.00	0.003	[34]
IL@CoFe_2_O_4_NPs@MWCNTs/GCE	0.02–160	0.0135	[35]
β-CDP/GCE	0.036–14.4	0.02	This work

**Table 2:** Recovery test for FNT in cabbage and water samples (*n* = 2).

Sample	Added (ng/mL)	Detected (ng/mL)	Recovery (%)
Cabbage 1	2000	2145	107.25
Cabbage 2	50	49.7	99.4
Water 1	2000	2240	112
Water 2	50	49.17	98.34

## Data Availability

The data presented in this study are available on request from the corresponding author. The data are not publicly available due to project confidentiality.

## Supplementary Materials

The following supporting information can be downloaded at: https://www.mdpi.com/article/10.3390/s23010435/s1, Figure S1: the scanning electron microscope images of bare GCE and β-CDP/GCE: (a) bare GCE; (b) β-CDP/GCE; Figure S2: the EIS measurements of bare GCE and β-CDP/GCE in 5.0 mM K_3_[Fe(CN)_6_]/K_4_[Fe(CN)_6_](1:1) containing 0.1 M KCl; Figure S3: (a) the second CV curves of FNT (2 μg/mL in acetate buffer solution, pH=5.00) with different scan rate (10, 50, 100, 200, 300 mV/s); (b) the plot of the *I*_pc,2_ against the scan rate (*v*); Figure S4: the electrochemical response of FNT (2 μg/mL in acetate buffer solution, pH = 5.00) with different methods: (black line) the first 2 CV curve with potential from 0.6 to −1.0 V and scan rate of 50 mV/s; (red line) the third-step DPV curve with potential increment of 13 mV, amplitude of 50 mV, pulse width of 60 ms, sampling width of 20 ms, pulse period of 500 ms and potential from 0.6 to −0.9 V; Figure S5: the third-step DPV curves of different electrodes in blank (no FNT) acetate buffer solution (pH = 5.00), DPV parameters: potential increment of 13 mV, amplitude of 50 mV, pulse width of 60 ms, sampling width of 20 ms, pulse period of 500 ms and potential from 0.6 to −0.9 V; Figure S6: the variation of *I*_pc,2_ with the accumulation time of every step of DPV method: (a) the first step; (b) the second step; (c) the third step; Figure S7: (a) the variation of *I*_pc,2_ with potential increment; (b) the variation of *I*_pc,2_ with amplitude.; Figure S8: (a) The *I*_pc,2_ of seven proposed sensors prepared in the same manner; (b) the variation of *I*_pc,2_ with different interfering compounds.
