# Supplementary material for: An Electrochemical Sensor Based on Electropolymerization of β-Cyclodextrin on Glassy Carbon Electrode for the Determination of Fenitrothion
Source: Sensors (Basel). 2022 Dec 30;23(1):435. doi: 10.3390/s23010435 (PMC9824020; doi:10.3390/s23010435)
Supplement: Supplementary file 1 [file sensors-23-00435-s001.zip › sensors-2072137-supplementary.pdf]

Supporting Information

An Electrochemical Sensor Based on Electropolymerization of β-Cyclodextrin on Glassy Carbon Electrode for the Determination of Fenitrothion

Rong Wang [1,†], Shulong Wang [1,†], Caihong Qin [1], Qiyang Nie [1], Yougang Luo [1], Qi-Pin Qin [1], Ruijuan Wang [1], Baiquan Liu [2,3,*] and Dongxiang Luo [4,*]

[1] Guangxi Key Lab of Agricultural Resources Chemistry and Biotechnology, College of Chemistry and Food Science, Yulin Normal University, 1303 Jiaoyudong Road, Yulin 537000, China
[2] State Key Laboratory of Luminescent Materials and Devices, South China University of Technology, 381 Wushan Road, Guangzhou 510640, China
[3] School of Electronics and Information Technology, Sun Yat-sen University, Guangzhou 510275, China
[4] Huangpu Hydrogen Innovation Center, School of Chemistry and Chemical Engineering, Guangzhou University, Guangzhou 510006, China
* Correspondence: liubq33@mail.sysu.edu.cn (B.L.); luodx@gzhu.edu.cn (D.L.)
† These authors contributed equally to this work.

1. The scanning electron microscope images of bare GCE and β-CDP/GCE

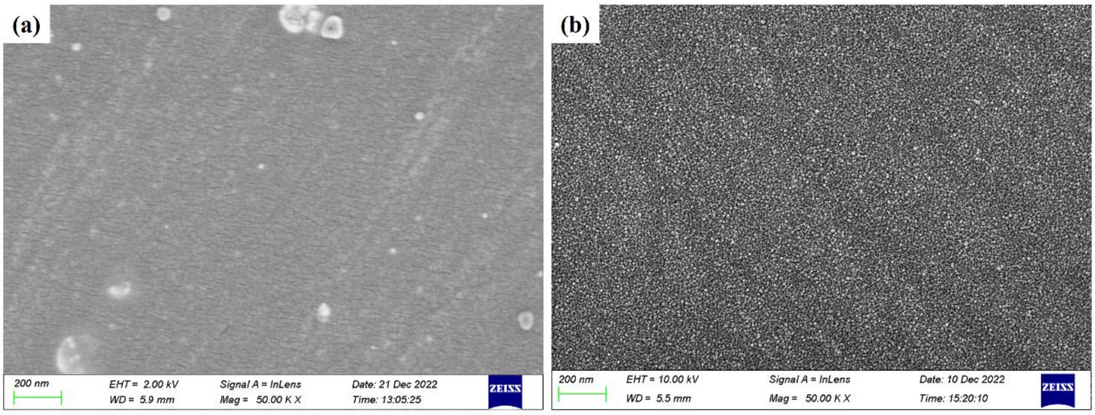

Figure S1. The scanning electron microscope images of bare GCE and β-CDP/GCE: (a) bare GCE; (b) β-CDP/GCE.

2. The EIS measurements of bare GCE and β-CDP/GCE

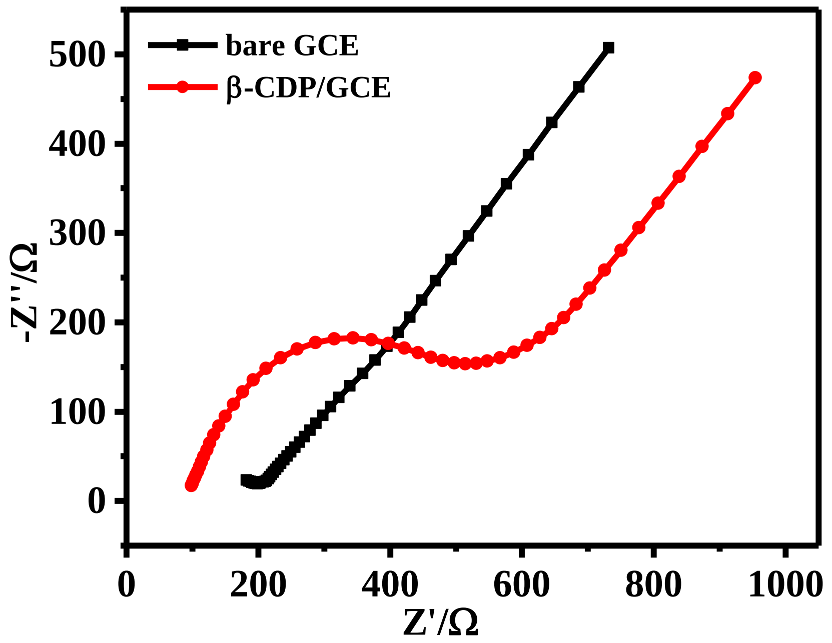

Figure S2. The EIS measurements of bare GCE and β-CDP/GCE in 5.0 mM $K_3[Fe(CN)_6]/K_4[Fe(CN)_6]$(1:1) containing 0.1 M KCl.

3. The effect of scan rate on the $I_{pc,2}$ at β-CDP/GCE

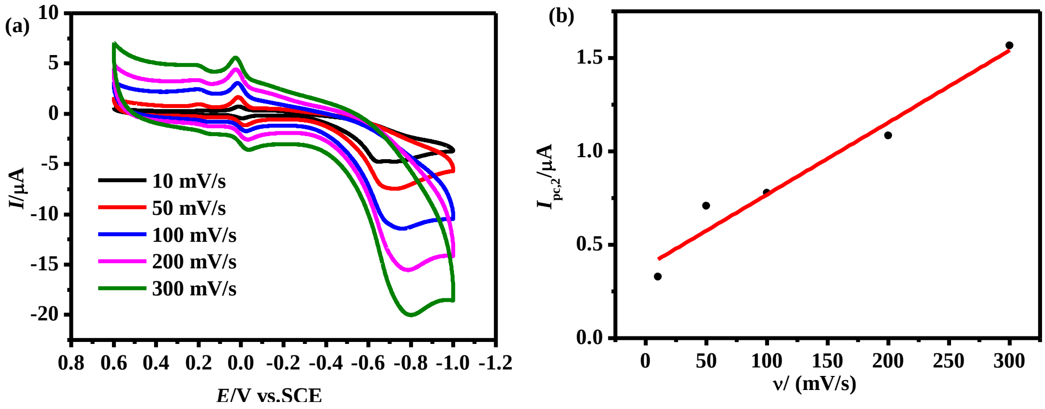

Figure S3. The second CV curves of FNT (2 μg/mL in acetate buffer solution, pH=5.00) with different scan rate (10, 50, 100, 200, 300 mV/s); (b) the plot of the $I_{pc,2}$ against the scan rate (v).

4. The electrochemical response of FNT with different methods

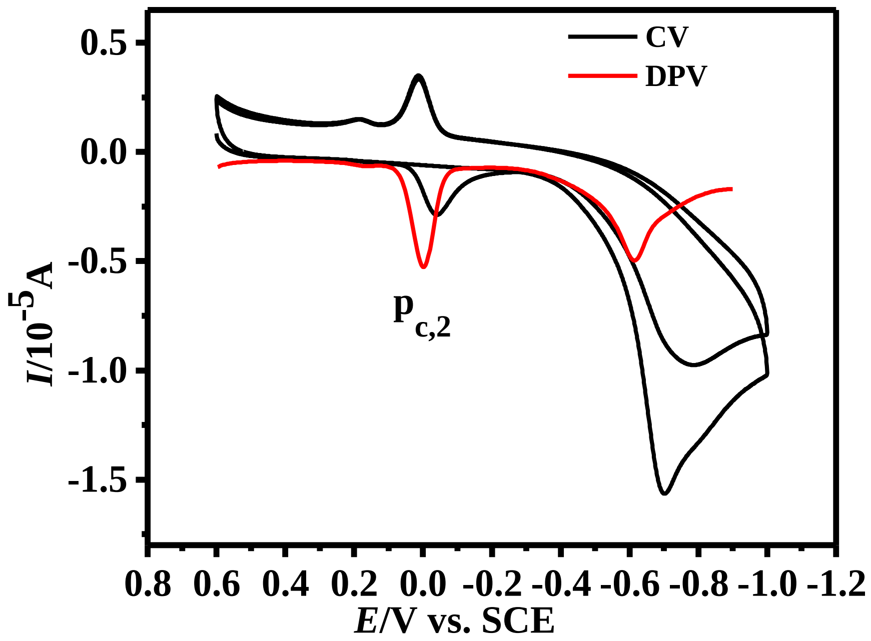

Figure S4. The electrochemical response of FNT (2 μg/mL in acetate buffer solution, pH=5.00) with different methods: (black line) the first 2 CV curve with potential from 0.6 to -1.0 V and scan rate of 50 mV/s; (red line) the third-step DPV curve with potential increment of 13mV, amplitude of 50mV, pulse width of 60ms, sampling width of 20ms, pulse period of 500ms and potential from 0.6 to -0.9 V.

5. The electrochemical behavior of β-CDP/GCE in blank acetate buffer solution

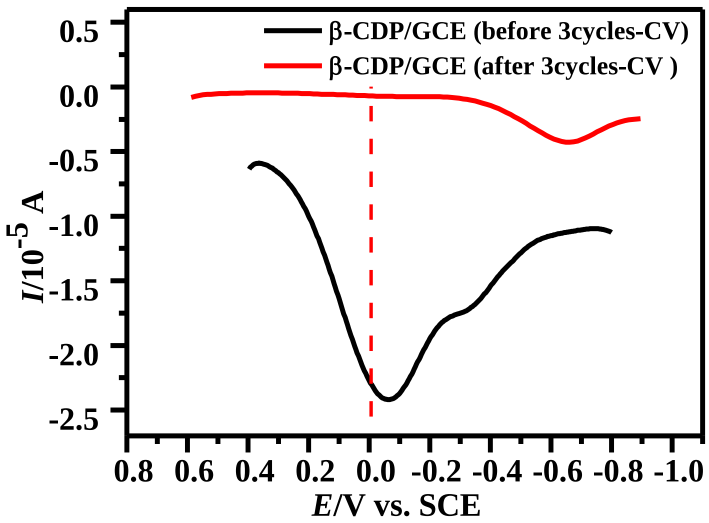

Figure S5. The third-step DPV curves of different electrodes in blank (no FNT) acetate buffer solution (PH=5.00), DPV parameters: potential increment of 13 mV, amplitude of 50 mV, pulse width of 60 ms, sampling width of 20 ms, pulse period of 500 ms and potential from 0.6 to -0.9 V

6. The variation of $I_{pc,2}$ with the accumulation time of every step of DPV method

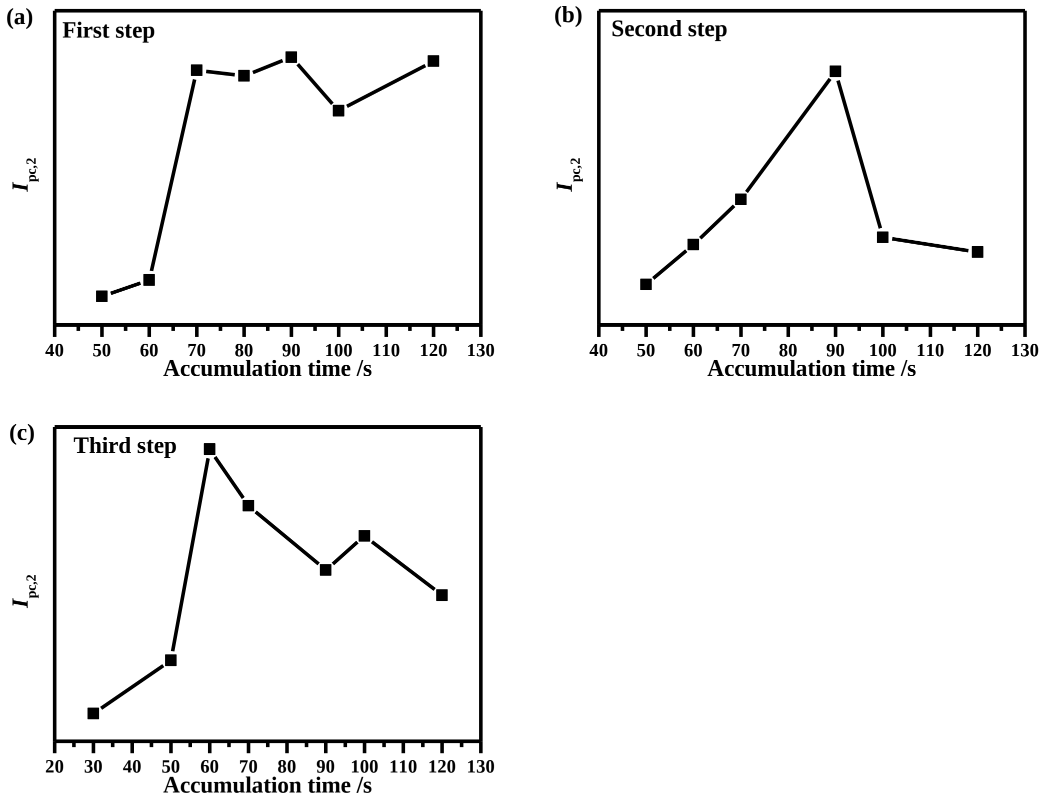

Figure S6. The variation of $I_{pc,2}$ with the accumulation time of every step of DPV method: (a) the first step; (b) the second step; (c) the third step.

7. The variation of $I_{pc,2}$ with potential increment and amplitude

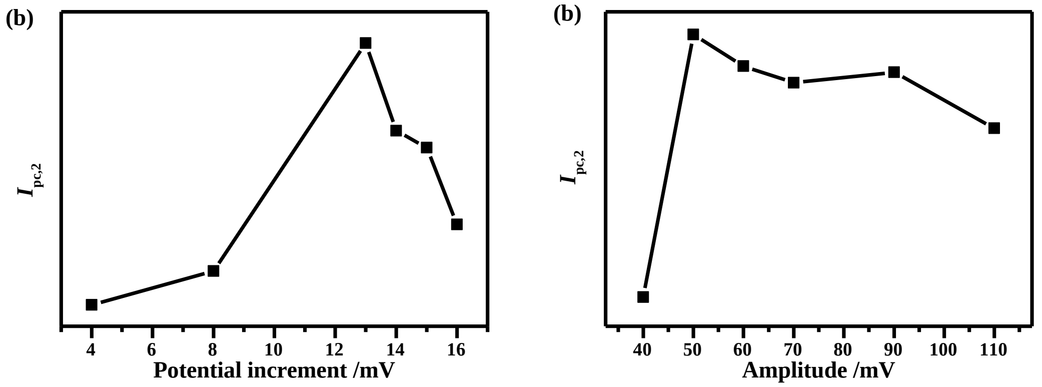

Figure S7. (a) The variation of $I_{pc,2}$ with potential increment; (b) the variation of $I_{pc,2}$ with amplitude.

8. Repeatability and interference

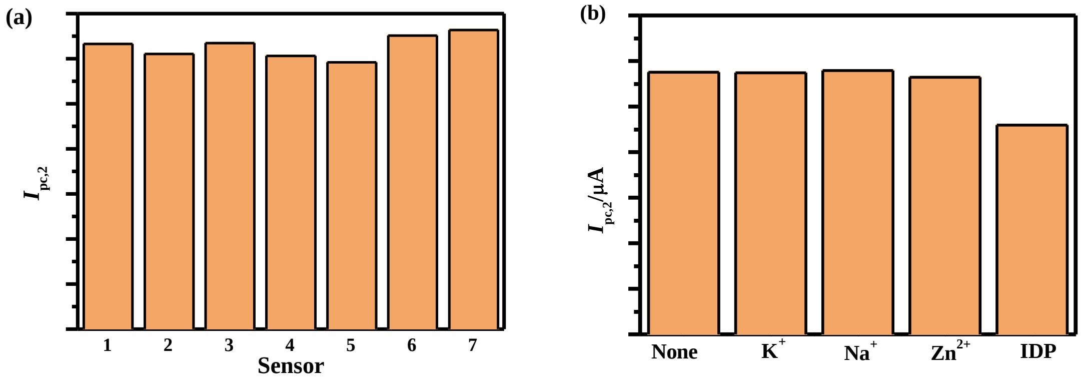

Figure S8. (a) The $I_{pc,2}$ of seven proposed sensors prepared in the same manner; (b) the variation of $I_{pc,2}$ with different interfering compounds.
